# Preparation Methods and Multifunctional Applications of Functionalized Electrospun Nanofibers for Biomedicine

**DOI:** 10.3390/nano15120909

**Published:** 2025-06-11

**Authors:** Jingwen Liu, Kai Wang, Fengying Jin, Jiayi Li, Yile Bin, Xiaofei Qian

**Affiliations:** 1 The First School of Clinical Medicine, Southern Medical University, Guangzhou 510515, China; 2 School of Microelectronics, Fudan University, Shanghai 200433, China; 3 Fudan Zhangjiang Institute, Shanghai 201203, China

**Keywords:** electrospinning, nanofibers, biomedicine, antibacterial, anti-inflammatory, tissue engineering, drug delivery, sensors

## Abstract

Electrospinning has emerged as a versatile and cost-effective technique for fabricating nanofibers with a high surface area, tunable morphology, and exceptional mechanical properties, demonstrating significant potential for applications in biomedicine. This review summarizes the main parameters of the electrospinning process and fabrication methods of functionalized electrospun nanofibers (FENFs) through one-step functionalization and post-functionalization. The applications of FENFs, with their antibacterial activity, anti-inflammatory effects, and tissue regenerative effects, as well as their potential in drug delivery systems and sensors, showcase their capability to address challenges in wound healing, cancer therapy, and health monitoring. Current limitations and future research directions are also identified. This review provides valuable insights for advancing research on nanofiber-based materials and their practical implementations.

## 1. Introduction

Electrospinning is a technique that utilizes a high-voltage electric field to stretch charged jets of polymer solutions or melt them into nanoscale and microscale fibers [1]. The development of electrospinning can be traced back to the 17th century. In 1600, William Gilbert was the first to observe the electrostatic attraction of liquids. In his 1628 treatise, Gilbert introduced the concept of electrospinning. This concept was derived from observations of electrostatic attraction, electric forces, and magnetic poles. In 1934, Anton Formhals patented the first method for producing nanofibrous materials from polymer solutions via electrospinning. Between 1964 and 1969, Geoffrey Taylor mathematically modeled the formation of conical droplets under an electric field, introducing the concept of the “Taylor cone”, which laid the theoretical foundation for electrospinning. However, for the next two decades, no significant advancements in electrospinning were reported in the literature [2,3]. It was not until the 1990s, with the rise of nanotechnology, that electrospinning gained widespread attention and experienced rapid development, leading to an exponential increase in published works in this field [4,5].

**Figure 1:**
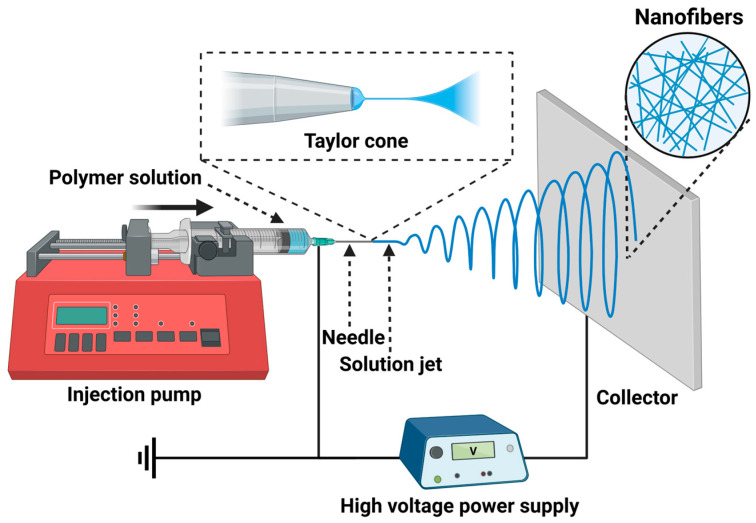
Schematic diagram of basic setup and process of electrospinning. Created in BioRender. Wang, K. (2025), https://BioRender.com/2vkwzzl (accessed on 1 June 2025).

As shown in Figure 1, electrospinning apparatus mainly consists of a high-voltage power supply system, a needle, an injection pump, and a ground metal collector [6]. During electrospinning, when a high voltage (about 5–60 kv) is applied between the tip of the spinneret and a metal collector, an electrical charge is generated on the surface of the polymer solution. As the solution droplet forms and approaches the tip of the spinneret, the charge accumulated on its surface causes the droplet to convert into a Taylor cone. Electrostatic repulsion is generated between similar charges in the polymer solution, resulting in Coulomb forces due to the applied electric field. When the electrostatic repulsion surpasses a critical value, the polymer solution is ejected from the tip of the Taylor cone and forms a polymer jet. Subsequently, the jet extends and the solvent evaporates while the jet solidifies, forming a nonwoven fibrous matrix due to the deposition of ultrafine fibers onto the collector [7,8,9,10].

Due to environmental protection concerns, bio-based polymers have been widely used in electrospinning due to their exceptional biodegradability and biocompatibility [11,12]. Based on their production methods and raw material sources, bio-based polymers can be classified into two major categories: synthetic and natural polymers [13]. These polymers typically exhibit different properties. Synthetic polymers present notable advantages in terms of synthetic versatility and precise structural control. Nevertheless, these materials frequently suffer from limited hydrophilicity and insufficient cellular recognition sites, leading to diminished cell affinity. On the other hand, natural polymers exhibit superior biocompatibility and low immunogenicity. Despite these benefits, the utilization of natural polymers is hindered by their poor processability, suboptimal mechanical properties, high solubility, and potential risks of disease transmission. Therefore, the combined use of synthetic polymers and natural polymers is a common strategy for fabricating high-performance electrospun nanofibers [14].

In recent years, electrospinning technology has emerged as a research hotspot in biomedicine due to its ability to fabricate nanofibers with high specific surface areas and tunable morphologies [9]. The unique structure of these nanofibers not only exhibits nanoscale size effects and surface characteristics but also provides excellent mechanical properties and flexibility [15]. Functionalized electrospun nanofibers (FENFs) are advanced materials prepared by integrating functional components (e.g., antibacterial agents, drugs, conductive materials, and photocatalytic materials) into nanofibers through electrospinning technology [16,17]. While inheriting the intrinsic properties of nanofibers, FENFs are endowed with specific physical, chemical, or biological functionalities through the incorporation of functional components. For instance, FENFs loaded with silver nanoparticles (Ag NPs) or antimicrobial peptides (AMPs) exhibit superior antibacterial properties [18,19]; FENFs incorporating drugs or growth factors can achieve controlled drug release and promote tissue regeneration [20]; and FENFs integrating conductive or photo-responsive materials can be utilized in flexible electronic devices or smart sensors [21,22].

This review provides a comprehensive summary of FENFs in terms of the key parameters of electrospinning technology, the two main preparation methods for functionalization, and the biomedical applications of FENFs with regard to their antibacterial activity, anti-inflammatory properties, and tissue repair abilities, as well as their potential in drug delivery, sensors, and other special applications. Additionally, this review also outlines the major challenges currently faced in the field and future development trends, in order to offer valuable insights and inspiration for future research on FENFs.

## 2. Preparation of Functionalized Electrospun Nanofibers

### 2.1. Main Parameters Affecting the Electrospinning Process

The large number of parameter options allows researchers to tune specific parameters to obtain the desired composition, size, and structure of electrospun nanofibers to increase their functionality for practical applications. They are mainly divided into the following three categories: solution parameters, process parameters, and ambient parameters (Table 1) [6,8,23]. However, these parameters display complex interactions that make the prediction of fiber morphology challenging. Therefore, it is crucial to clarify and summarize how the key parameters influence the final outcomes.

#### 2.1.1. Solution Parameters

##### Polymer Concentration

Sufficiently high polymer concentration is a prerequisite for electrospinning as it leads to adequate chain overlap and entanglement, which allow for continuous fiber extension [24,25,26]. Increased concentration elevates surface tension, viscosity, mechanical strength, and fiber diameter [27]. For example, in a study by Liu et al., when a series of polyacrylonitrile (PAN) solutions with different concentrations (3.5 wt.%, 6.0 wt.%, and 8.0 wt.%) were used for electrospinning the fiber diameter increased with concentration while remaining at the nanoscale [28].

However, the concentration of the polymer solution must be maintained within an optimal range. Excessive concentrations hinder the solution mobility propelled by the electric field, causing needle tip clogging and yielding beaded or defective fibers. Conversely, insufficient concentrations prevent adequate chain entanglement, resulting in fiber breakage and the formation of beaded structures or discontinuous fibers during electrospinning [29].

##### Molecular Weight of Polymers

Polymer molecular weight, characterizing chain length, governs chain entanglement and modulates solution viscosity and surface tension [26]. At a constant concentration, higher molecular weights facilitate continuous fiber formation by promoting chain entanglement and stabilizing the electrospinning jet, whereas lower molecular weights result in bead formation [25,29,30]. Vigani et al. improved the electrospinnability of sodium alginate (ALG) by blending high-molecular-weight (h-PEO, 4000 kDa) and low-molecular-weight (l-PEO, 600 kDa) polyethylene oxide (PEO). The incorporation of h-PEO significantly increased chain entanglement density, facilitating the electrospinning process, but resulted in microfibers. In contrast, the use of l-PEO reduced the fiber diameter from the micrometer range (13.3 ± 1.9 μm) to the nanoscale (213 ± 5 nm). Furthermore, the introduction of h-PEO enhanced the mechanical strength of the fibers while maintaining nanoscale dimensions (253 ± 11 nm) [31].

##### Viscosity

Viscosity, as an important parameter of rheology, is mainly used to describe the resistance of a fluid or material to shear forces during flow. It is determined by the molecular weight, molecular weight distribution, structure, and concentration of the polymer as well as the nature of the solvent [32,33]. Several studies have shown that the underlying cause of fiber morphology is viscosity rather than concentration [34]. In fact, viscosity, concentration, and molecular weight are related to each other; for example, viscosity increases with concentration [29]. Within the optimal viscosity range, enhanced viscosity suppresses bead formation and yields uniform, smooth nanofibers with increased diameters [25,34,35]. Insufficient viscosity amplifies surface tension effects, promoting bead formation and hindering continuous fiber production [30]. Chi’s study revealed that using polyvinyl alcohol (PVA) as a thickening agent significantly influences the viscosity of mixed solutions. The pure PVA solution (12 wt.%) exhibited a remarkably high viscosity of 4300.33 centipoises (cP), while the pure gelatin solution showed a much lower viscosity of merely 12.5 cP. When these two components were mixed at different volume ratios the viscosity increased exponentially with the PVA proportion [36].

The storage (G’) and loss (G”) moduli, combined with viscosity, determine the viscoelastic and viscoplastic properties of polymer solutions, serving as key rheological parameters. Although G’ is necessary for jet initiation, excessive G’ promotes jet shrinkage, hindering elongation and potentially causing jet breakage and droplet formation. Conversely, adequate G” enables continuous jet stretching toward the collector [30]. Elevated polymer concentrations simultaneously increase both the storage (G’) and loss (G”) moduli while decreasing the G’/G” ratio as a whole [37].

##### Conductivity

Solution conductivity significantly influences electrospinning dynamics. Increased solution conductivity facilitates enhanced jet extension in the electrospinning process within limits, while excessive conductivity induces jet instability and bead formation [25,29,35]. Polymer solution conductivity, which is intrinsically linked to charge density, is governed by polymer properties, solvent characteristics, and ionic additives [26,29].

Polymer properties: Natural polymers like chitosan typically exhibit polyelectrolytic behavior with pH-dependent conductivity, whereas synthetic polymers like poly(lactic-co-glycolic acid (PLGA) often require elevated voltages to compensate for their inherent low conductivity [38].

Solvent characteristics: Higher solution dielectric constants promote greater charge accumulation and electrostatic repulsion, enabling jet splitting at the needle tip and the subsequent formation of finer nanofibers [30].

Ionic additives: Incorporating salts or ionic components enhances solution charging; Herrero et al. improved PLGA solution conductivity and spinnability through the addition of pyridine, which spontaneously evaporated during electrospinning, obviating post-processing removal [39].

##### Surface Tension

Due to Rayleigh instability, surface tension typically converts a liquid jet into one or more spherical droplets to reduce the surface area. Polymer solutions with high surface tension are more likely to produce defective fibers [29,39]. Therefore, the question of how to reduce the surface tension to produce continuous fibers is commonly considered by researchers in pre-experiments [33,35]. For instance, Vítková et al. [40] significantly enhanced the electrospinnability of hyaluronic acid (HA)/PVA-blended solutions by incorporating benzalkonium chloride (BEC) as a surfactant. Their study demonstrated that with increasing BEC content (from 0.006 wt.% to 0.065 wt.%) the solution’s surface tension decreased from 43.7 mN·m^−1^ to 41.1 mN·m^−1^, while the conductivity increased from 1473 µS·cm^−1^ to 1546 µS·cm^−1^. These modifications facilitated fiber formation and resulted in a gradual reduction in fiber diameters from 0.1–0.5 µm to 0.03–0.1 µm.

#### 2.1.2. Process Parameters

##### Applied Voltage

Applied voltage significantly influences electrospun nanofibers’ diameter, though this relationship remains debated due to conflicting experimental observations. Tuz Zahra et al. [25] demonstrated that increasing voltage initially reduces polyvinylpyrrolidone (PVP) fiber diameter, but excessive voltage causes fluid over-ejection, yielding thicker fibers. The average diameters were observed to be 0.270 ± 0.086 µm, 0.233 ± 0.079 µm, and 0.255 ± 0.063 µm for 15 kV, 20 kV, and 25 kV applied voltages, respectively. Herrero et al. [39] observed that the fiber diameter initially increased and then decreased with increasing applied voltage, reaching a maximum value at intermediate voltages of around 15 kV before declining (the average diameters were 1.51 µm at 13 kV, 1.76 µm at 15 kV, and 1.62 µm at 17 kV).

In fact, the relationship between applied voltage and the diameter of electrospun fibers is multifactorial. Higher voltage not only increases the solution charge and electrostatic repulsion, potentially reducing diameter, but also simultaneously expands the size of the Taylor cone, promoting fluid ejection [25,27,29,33]. Thus, the final diameter reflects a balance between these competing effects.

##### Flow Rate

Optimal flow rate selection is crucial for successful electrospinning and achieving desired fiber properties. In general, an increase in the flow rate results in larger fiber diameters. Nevertheless, insufficient flow rates prevent Taylor cone formation, while excessive flow rates cause instability of the Taylor cone and subsequent jet stretching [25,27,29]. The ratio of the flow rate to the applied voltage should be taken into account as it defines the amount of polymer supplied to the jet that is acceptable for the flow rate provided by the electric field [26].

##### Collecting Distance

Extended distance prolongs the process of jet stretching, reducing fiber diameter through increased elongation [26]. Conversely, insufficient distances limit drying time, causing fiber non-uniformity or bead formation. Optimal distance selection must balance adequate flight time for uniform fiber production with practical constraints. In fact, these three process parameters exhibit interdependent relationships. In Najim et al.’s study, under constant conditions of 20 kV voltage and a 1.5 mL/h flow rate, increasing the collecting distance from 15 cm to 20 cm reduced the average fiber diameter from 345.4 ± 102 nm to 218.4 ± 78 nm. When maintaining a fixed collecting distance of 20 cm, elevating the voltage from 18 kV to 25 kV decreased the fiber diameter from 221.8 ± 62 nm to 149.8 ± 58 nm. However, exceeding the critical voltage threshold (25 kV) induced jet instability, resulting in occasional bead defects, which necessitated synergistic optimization with the flow rate (1 mL/h) for optimal results [41].

#### 2.1.3. Ambient Parameters

##### Temperature

Temperature significantly influences electrospinning, mainly through its effects on solvent evaporation and solution viscosity. Elevated temperatures accelerate solvent evaporation while reducing solution viscosity, collectively impacting nanofiber morphology [33]. Ideal temperature ranges allow sufficient time for jet splitting and elongation during flight, followed by complete solvent evaporation upon reaching the collector, and finally yielding minimal fiber diameters. However, overly high temperatures induce premature solvent evaporation, limiting jet stretching, whereas inadequate temperatures hinder complete solvent volatilization, collectively leading to larger fiber diameters and wider size distributions [32].

##### Relative Humidity

Relative humidity (RH) significantly influences nanofiber morphology and porosity through solvent evaporation modulation [33,42].

The effect of RH on fiber diameter is highly dependent on the polymer and solvent systems; for example, high RH generally delays solvent evaporation, extending jet stretching time. This prolonged process yields fine fibers with significant diameter heterogeneity while sustaining elevated polymer concentrations, which enhances elastic forces (G’) over viscous forces (G”), consequently promoting bead formation [34,37,43]. However, the opposite situation has also been reported, where the diameter of electrospun cellulose acetate (CA) and polystyrene (PS) fibers increased with increasing RH as water changed the nature of the polymer solution [44].

Vapor-induced phase separation (VIPS) serves as a principal method for creating porous membranes through electrospinning. This process involves non-solvent vapor (typically water) interacting with and permeating the polymer jet, causing polymer precipitation to form a solid matrix while the solvent-rich phase generates porous structures. VIPS predominantly occurs under high-RH conditions, where enhanced water–solution interdiffusion promotes phase separation [34,44]. While VIPS represents a primary porosity mechanism, alternative processes, including breath figure self-assembly, thermally induced phase separation (TIPS), and non-solvent induced phase separation (NIPS), also contribute to membrane porosity [43,45].

### 2.2. Functionalization Methods of Electrospun Nanofibers

**Figure 2:**
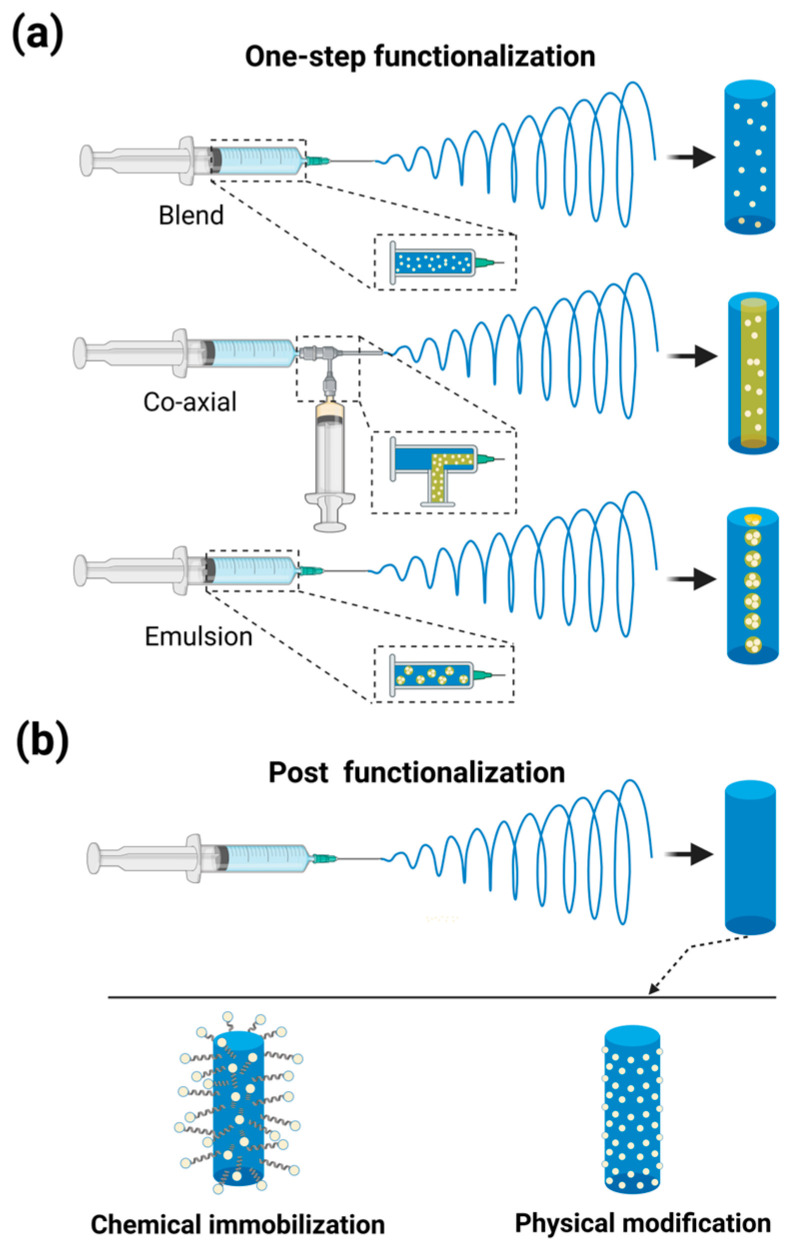
The two main methods for incorporating functional components into electrospun fibers. (**a**) One-step functionalization primarily involving blend electrospinning, co-axial electrospinning, and emulsion electrospinning techniques. (**b**) Post-functionalization, which is a two-step process: first, the fiber matrix is obtained by electrospinning, and then the surface is modified with functional components by chemical immobilization or physical modification methods. Created in BioRender. Wang, K. (2025), https://BioRender.com/u0t70e5 (accessed on 1 June 2025).

Functionalized nanofibers represent a cutting-edge class of nanomaterials that combine the unique physical properties of nanofibers with tailored functionalities, enabling a wide range of advanced applications, including in antibacterial dressings, drug delivery, tissue engineering, and energy transformation [17]. Typically, there are two main approaches to incorporating functional components into electrospun nanofibers (Figure 2): (i) one-step functionalization, where polymers and functional components are directly electrospun together using techniques such as blend, co-axial, and emulsion electrospinning; and (ii) post-functionalization, which involves a two-step process wherein the fiber matrix is first obtained through electrospinning, followed by the modification of the surface with functional components via chemical immobilization or physical modification methods [46,47].

#### 2.2.1. One-Step Functionalization

Among these methods, blend electrospinning stands out as the simplest approach for fabricating functionalized nanofibers. This is primarily due to its straightforward requirement of dissolving both the fiber-forming polymer and the functional components within the same solvent system, utilizing the most basic electrospinning apparatus. In this process, each part should be dispersed uniformly within the polymer solution and the resultant nanofibers [48]. Therefore, the compatibility and interfacial tension between polymers, as well as between polymers and functional components, should be carefully considered. The addition of compatibilizers to reduce interfacial tension between phases is a commonly employed strategy. For instance, hydroxyapatite (HA), an osteoconductive bioceramic, requires strong interfacial adhesion/affinity with polymer matrices to achieve the structural and mechanical integrity of the final composite material. However, most biodegradable polymers cannot form sufficient interfacial bonds with HA. Kutikov et al. [49] addressed this issue by incorporating hydrophilic polyethylene glycol (PEG) blocks with poly(D,L-lactic acid) to create an amphiphilic triblock copolymer, PLA-PEG-PLA (PELA). At 25 wt.% this copolymer reduced the interfacial tension in HA-PELA suspensions and improved electrospinnability. Alternatively, the co-solvent method, which involves using solvents capable of dissolving both polymers simultaneously, can achieve miscibility even between inherently immiscible polymers. For example, although polyvinyl chloride (PVC) and polyacrylonitrile (PAN) are chemically incompatible, both can be dissolved in dimethylformamide (DMF). Experimental results demonstrated that a PVC/PAN ratio of 25:75 was optimal, producing more stable nanofibers at low concentrations with improved compatibility between the two polymers [50].

Another facile strategy for fabricating functionalized nanofibers with a core–shell structure is co-axial electrospinning. This process involves ejecting two types of solutions through a spinneret composed of two co-axially aligned needles under a stable electrostatic field. Consequently, composite polymer droplets are formed at the spinneret, with the inner (core) liquid being pumped through the inner needle and the outer (shell) material delivered through the outer needle [51]. Similarly, the compatibility and interfacial tension of polymer solutions are critical factors determining the structural integrity of core–shell architectures. One study reveals that the key to successful co-axial electrospinning lies in maintaining a well-defined core–shell interface while ensuring constant and minimal interfacial energy at this boundary. Ideally, core and shell materials should exhibit partial miscibility. Complete immiscibility generates relatively high interfacial tension, potentially causing core rupture or even preventing the core material from entering the fiber-forming jet. Conversely, excessive miscibility eliminates the distinct interface, effectively converting the core into a coagulation bath for the shell solution and leading to premature gelation in the Taylor cone. Furthermore, the pre-electrospinning addition of a small amount of shell solvent into the core solution may help mitigate localized interfacial tension variations [52].

Emulsion electrospinning is another innovative technique for fabricating core–shell structures. It involves electrospinning a homogeneous blend of two or more immiscible liquids using a traditional single needle. This process relies on phase separation between the continuous phase and the droplet phase/dispersed phase under the influence of the electric field during electrospinning, where the continuous phase transforms into the fiber shell and the droplet phase forms the fiber core. Although this approach eliminates the requirement for a single-solvent system for all components, it typically requires more complex process conditions and the addition of suitable stabilizers, such as emulsifiers/surfactants (e.g., Tweens, Spans), Pickering particles (e.g., nano silica, nano clay), or biopolymers (e.g., soy proteins, whey proteins) [48]. This method minimizes functional components’ exposure to organic solvents, lowering molecular deactivation risks. It works well with both hydrophilic and hydrophobic polymers and can produce uniform core–shell structures without specialized equipment [53].

Core–shell structures have significant applications in drug delivery systems, where the shell layer acts as a barrier to effectively mitigate or eliminate the initial burst release, protect sensitive drugs from environmental degradation, and enable sustained and controlled drug release [54,55,56]. For instance, Su et al. [57] reported the loading of two different growth factors onto poly(L-lactide-co-caprolactone) (PLLACL)–collagen (3:1) nanofiber mats with a core–shell structure. In this system, dexamethasone (DEX) loaded in the shell exhibited a sharp initial burst release, while bone morphogenetic protein 2 (BMP2) loaded in the core showed a slow and stable long-term release.

#### 2.2.2. Post-Functionalization

Post-functionalization is an effective method for introducing functional components onto the surface of nanofibers after electrospinning. It is particularly suitable for incorporating functional components that are sensitive to high voltage and organic solvents. This approach avoids the loss of functionality of additives during the electrospinning process while preserving the original degradation rate and mechanical properties of the polymer matrix. Specific post-functionalization methods can be broadly categorized into two types: (i) chemical immobilization (e.g., plasma treatment and surface graft polymerization) and (ii) physical modification (e.g., non-covalent adsorption or solidification on the surface) [46,58].

By forming stable chemical bonds between the target molecules and the support material, chemical immobilization prevents leaching and ensures long-term functionality. Among these methods, plasma treatment has been demonstrated to enhance the biocompatibility of electrospun nanofibers and the release rate of functional components by altering surface hydrophilicity and chemical composition [59,60]. In a study by Cassan et al. [61], chitosan (CS) was introduced onto the surface of polycaprolactone (PCL) nanofibers through grafting and crystallization. The cationic surface charge resulting from the amino groups of chitosan allows for the attachment of anionic organic or inorganic drug delivery systems via electrostatic interactions.

By immobilizing functional components on the fiber surface, slow and sustained release can be achieved, thereby preserving their bioactivity and extending functional duration. Farkas et al. [62] adsorbed doxycycline (Doxy) onto a polylactic acid–hydroxyapatite (PLA-HA) carrier by dissolving Doxy at different concentrations in distilled water and stirring in a closed environment at room temperature. The results indicated that PLA-HAP-Doxy is a promising biomedical membrane, and its drug release performance can be optimized by adjusting the mass fraction of Doxy. Another study incorporated silver nanoparticles (Ag NPs) into PLA fabrics using two methods: pre-solution blending and post-solution casting (placing the electrospun membrane in a fixture and adding a silver colloid solution dropwise onto the surface, followed by drying). The antibacterial efficacy was evaluated against various bacterial strains, with the post-solution casting method demonstrating the most effective antibacterial properties [63].

## 3. Biomedical Applications of Functionalized Electrospun Nanofibers

**Figure 3:**
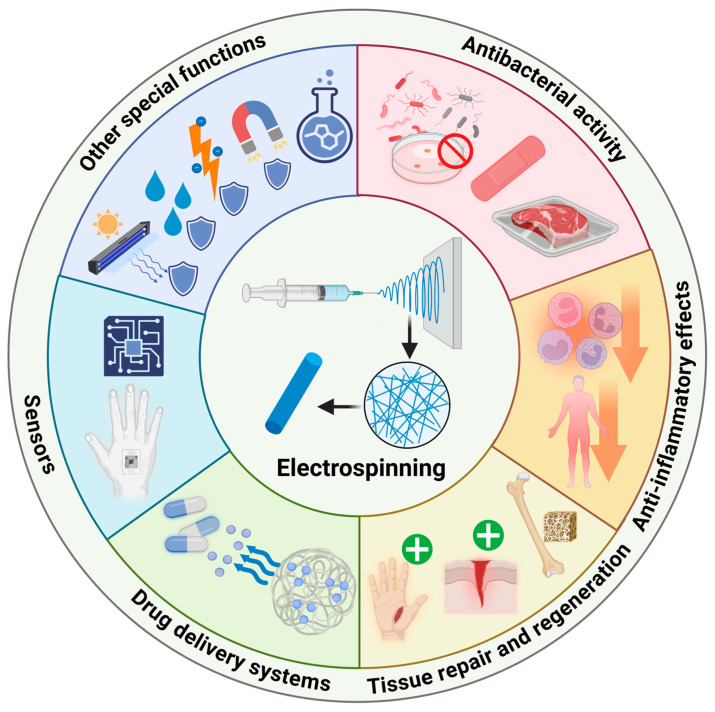
Main functional applications of electrospun nanofibers. Created in BioRender. Wang, K. (2025), https://BioRender.com/lkcznsi (accessed on 1 June 2025).

By incorporating specific functional materials, researchers can endow electrospun nanofibers with diverse functionalities [17,64,65]. In the light of current research trends, this review focuses on the advancements in the biomedical application of functionalized electrospun nanofibers in the following areas (Figure 3): (i) antibacterial activity, (ii) anti-inflammatory effects, (iii) tissue repair and regeneration, (iv) drug delivery systems, (v) sensors, and (vi) other special functions.

### 3.1. Antibacterial Activity

The antibacterial materials loaded in electrospun nanofibers are the key factors that determine their effectiveness.

Based on their chemical composition and origin, antibacterial materials can be categorized into different groups, primarily including traditional antibacterial agents, nanomaterials, nature-derived compounds, and biofunctional agents [66]. Antibacterial agents refer to drugs that possess bactericidal or bacteriostatic activity, encompassing both antibiotics and various chemically synthesized drugs, which are commonly used in the clinical treatment of bacterial infections. However, their misuse and overuse contribute to the emergence of multidrug-resistant (MDR) microorganisms [67,68]. Nanomaterials are generally categorized into the following three types: inorganic (e.g., Ag NP, TiO_2_ NP, ZnO NP, MXene, GO), organic (e.g., CS NP, COFs), and inorganic–organic hybrids [46,69]. Inorganic nanomaterials typically offer good stability, high thermal resistance, and chemical resistance, whereas organic nanomaterials may provide better biocompatibility and processability [70]. Hybrid nanomaterials combine these advantages, enabling enhanced stability, mechanical performance, and synergistic bactericidal effects [71]. Notably, metal–organic frameworks (MOFs) like ZIF-8 represent prominent hybrid systems [72]. Nature-derived compounds mainly include plant extracts and animal extracts [73,74]. Plant extracts typically consist of natural active ingredients derived from roots, stems, or leaves, such as curcumin (CUR) [75], chlorogenic acid (CA) [76], and essential oils rich in phenols or terpenes [77]. Certain natural secretions and liquid extracts from animals, such as honey, have also demonstrated excellent antibacterial properties [78]. Biofunctional agents currently under extensive research include a new generation of antibacterial agents that have emerged in recent years, known as antimicrobial peptides (AMPs) [69,79]. AMPs primarily feature cationic and amphipathic regions with an α-helical conformation, which facilitate interaction with the negatively charged bacterial surfaces [47].

As shown in Table 2, electrospun nanofibers with antibacterial activity play a significant role in promoting human health [80], particularly in the fields of wound dressings [81] and food packaging [82].

Electrospun nanofibers have emerged as a promising platform for wound dressing applications due to their large surface area and high porosity. Christodoulou et al. compared the efficacy of linear PLA and star-shaped PLA–pentaerythritol (PLA-PE) copolymers as carriers for the transdermal delivery of the antibacterial agent levofloxacin (LEV). Compared to PLA (82.4%), PLA-PE nanofibers exhibited smaller diameters and higher porosity (90.1%), resulting in an enhanced drug loading capacity (34.78%) and superior application potential. In fact, the properties of functional antibacterial materials exhibit complex interactions with electrospun nanofiber performance. Velu et al. found that flufenamic acid (FFA) dispersed within the amorphous matrix of chitosan/polyvinyl alcohol (CHS/PVA) nanofibers enhanced drug stability. Conversely, the incorporation of FFA significantly reduced nanofiber diameters. In another approach, Esfanjani et al. developed PCL electrospun nanofibers coated with gum tragacanth (GT)-containing thymol (Thy)-loaded layered double hydroxide (LDH) nanohybrids, which enabled sustained thymol release. The results demonstrated that LDH/Thy incorporation markedly improved the composite’s hydrophilicity. Notably, adding 10% LDH to PCL/GT nanofibers increased their tensile strength from 0.27 MPa to 1.74 MPa. The PCL/GT/LDH/Thy nanofibers exhibited superior antibacterial activity against *E. coli*, *S. aureus*, and *MRSA*, correlating with sustained Thy release (78% over 110 h). Compared to controls, the nanofibers significantly enhanced wound healing rates (from 80.33% to 48.5%, respectively).

Electrospinning technology has been widely adopted as an innovative strategy for developing biodegradable active packaging (AP). In a study by Alam et al., hybrid electrospun gelatin (HENF) AP was fabricated using 30% gelatin (GE) combined with 1%, 2%, and 3% green tea extract powder (GTEP) (designated as HGGTNF) and applied to Hanwoo beef. Compared with traditional polyethylene packaging (PEP), HGGTNF significantly maintained the physicochemical properties of the food product, including pH, oxidative stability, and color. More importantly, the HGGTNF 3% sample demonstrated remarkable reductions in total coliform count (TCC) (0.74 ± 0.04 log CFU/g), total viable count (TVC) (1.38 ± 0.05 log CFU/g), and total yeast and mold count (TYMC) (1.59 ± 0.06 log CFU/g), indicating effective antimicrobial efficacy. In another study by Wang et al., a novel packaging film for aquatic products was developed via electrospinning using quaternized chitosan, polyvinyl alcohol, and thymol. The incorporation of thymol endowed the nanofiber membrane with excellent antibacterial and antioxidant properties. With increasing voltage, the specific surface area and pore area of the nanofiber membrane first increased and then decreased. At an applied voltage of 20 kV, the electrospun nanofiber membrane exhibited the highest adsorption rate of 78.28%, significantly enhancing its efficiency in capturing fishy odors.

### 3.2. Anti-Inflammatory Effect

Inflammation is a complex network involving multiple cytokines, cells, and pathways [104,105]. Inflammatory responses play a crucial role in local immune reactions and determine the progression of wound healing [106]. Under normal circumstances, the protective and destructive effects of the inflammatory cascade are in a dynamic balance. However, if inflammatory triggers persist or the normal healing process is disrupted, acute inflammation may not resolve but instead progress to chronic inflammation, which can lead to numerous diseases, such as rheumatoid arthritis, atherosclerosis, multiple sclerosis, inflammatory bowel disease, and cancer [105,107].

Among the various inflammatory cells, macrophages play a central role in the inflammatory response and have been extensively studied in immunomodulation research. Macrophages are highly plastic cells capable of transitioning between two main subtypes: M1 and M2. M1 macrophages secrete pro-inflammatory cytokines and exhibit potent antibacterial and antitumor activities, while M2 macrophages secrete anti-inflammatory cytokines and promote tissue repair and wound healing [108,109]. The timely transition of M1 macrophages to M2 macrophages is critical for restoring tissues to their normal state [106].

Given the complexity of the inflammatory environment, the latest research has demonstrated diversified anti-inflammatory strategies, frequently integrating multiple functions into electrospun nanofibers beyond anti-inflammation, such as antibacterial, antioxidant, immunomodulatory, and angiogenic effects [110]. This review broadly categorizes these strategies into three types, as indicated in Table 3: (i) direct strategies, which involve loading anti-inflammatory agents to modulate inflammatory cells (mainly macrophages) and cytokines in the inflammatory microenvironment; (ii) indirect strategies, which address the root causes of inflammation, such as eliminating various forms of pathogens or foreign bodies by antibacterial materials, as demonstrated above; and (iii) synergistic strategies, which involve the combined application of direct and indirect strategies.

### 3.3. Tissue Regeneration and Repair

Human tissues typically exhibit limited regenerative capacity after resection or injury, leading to functional loss and complex esthetic defects that significantly impact patients’ quality of life [16].

Tissue engineering (TE), a method for producing patient-specific tissue constructs to repair and replace damaged tissues, has garnered significant attention in the research community [126]. The key components required for TE are scaffolds, regenerative cells, and bioactive molecules [15,127]. Scaffolds play a central role, functioning similarly to the extracellular matrix (ECM) by providing biochemical (e.g., growth factors and collagen) and biophysical cues (e.g., fiber structure, hydrophilicity, and stiffness) for cell adhesion, proliferation, differentiation, and migration [128].

Electrospun nanofibers are the most widely used materials to construct scaffolds in TE due to their high porosity, high surface area-to-volume ratio, and superior mechanical properties [129,130]. The high porosity of scaffolds is crucial, as it not only provides the necessary space for cell migration, attachment, and proliferation but also facilitates oxygen and nutrient delivery and the removal of metabolic waste [16]. As demonstrated in Table 4, functionalized electrospun nanofiber scaffolds have been extensively explored in the fields of skin, bone, nerve, vascular, cartilage, and tendon tissue engineering.

The skin, the body’s largest protective organ, shields us from pathogen invasion and excessive water loss. Skin damage leads to the loss of barrier function, posing a serious threat to human health [131]. To promote epidermal regeneration, Echeverria et al. [132] fabricated scaffolds using PCL/sodium alginate (SA) solutions via electrospinning. Sodium ions in the alginate were then replaced with calcium ions to form PCL/calcium alginate (CA) scaffolds. The release of calcium ions from the PCL/CA scaffolds into the biological medium stimulates keratinocyte differentiation, enhancing full-thickness skin tissue regeneration.

Bone defects, which are commonly caused by tumors, trauma, osteoporosis, and infections, are among the most frequent issues in orthopedics. In most cases, the repair process for bone defects is complex and difficult to achieve naturally [133]. Barbosa et al. [134] created piezoelectric poly(vinylidene fluoride-co-trifluoroethylene) (PVDF-TrFE) nanofiber scaffolds filled with hydroxyapatite (Hap), successfully incorporating natural piezoelectric properties into electrospun nanofiber scaffolds for bone induction. The results demonstrated high ALP activity, calcium deposition, and osteogenic gene expression levels, highlighting the scaffold’s potential in bone tissue engineering.

For tissues with specific orientations or structures, such as blood vessels, nerves, and tendons, the correct spatial arrangement of tissue engineering scaffolds is crucial [135]. Blood vessels have a multilayered structure, where the intima and media layers are oriented perpendicularly and essential for maintaining vascular function. Guo et al. [136] used sequential electrospinning combined with folding and rolling operations to fabricate a three-layer vascular scaffold with perpendicularly oriented inner and middle layers. This scaffold effectively mimics the natural multilayered structure of blood vessels and has great potential for guiding the spatial arrangement of corresponding cells in vascular tissue. In a study on nerve tissue engineering by Zamanifard et al. [137], polyhydroxybutyrate (PHB) was electrospun and aligned nanofibers were collected using a rotating collector. To enhance scaffold functionality, post-processing techniques such as crosslinking, plasma treatment, coating, and inkjet printing were employed to introduce laminin, which promotes nerve cell growth, and conductive polyaniline (PANI) nanoparticles into the scaffold.

**Table 4:** Electrospun nanofibers which are used in tissue regeneration and repair.

Electrospun Polymers	Functional Additives	Application Area	Ref.
Poly(ε-caprolactone) (PCL)/sodium alginate (CA)	Calcium chloride (CaCl_2_)	Skin tissue engineering	[132]
Pectin (Pec)/polyacrylic acid (PAA)	Platelet rich fibrin (PRF)/Simvastatin (SIM)	Skin tissue engineering	[138]
Poly(vinylidene fluoride-co-tetrafluoroethylene) (PVDF-TrFE)	Hydroxyapatite (HAp)	Bone tissue engineering	[134]
Polyvinyl alcohol (PVA)	Tetraethyl orthosilicate (TEOS)	Bone tissue engineering	[139]
Poly(ε-caprolactone) (PCL)	Hydroxyapatite (HAP)/roxithromycin (ROX)	Bone tissue engineering	[140]
Polylactide (PLA)	Graphene oxide (GO)/parathyroid hormone (rhPTH(1–34))	Bone tissue engineering	[141]
Polyvinylidene fluoride-trifluoroethylene (P(VDF-TrFE)/poly(N-isopropylacrylamide) (pNIPAM)	Barium titanate piezoelectric nanoparticles (BTNPs)/nerve growth factor (NGF)	Nerve tissue engineering	[142]
Polylactic acid (PLA)/polyaniline (PAni)	-	Nerve tissue engineering	[143]
Polyhydroxybutyrate (PHB)/gelatin (Gel)	Laminin/polyaniline nanoparticles	Nerve tissue engineering	[137]
Silk fibroin (SF)	Vascular endothelial growth factor (VEGF)/transforming growth factor (TGF) inhibitor	Vascular tissue engineering	[144]
Polycaprolactone (PCL)	Bioactive glasses (BGs)	Vascular tissue engineering	[145]
Poly-D,L-lactide-co-glycolide (PLGA)	Small intestine submucosa (SIS)	Tendon tissue engineering	[146]
Poly(butyl cyanoacrylate) (PBCA)	Copper oxide nanoparticles (CuO NPs)/caseinphosphopeptides (CPP)	Tendon tissue engineering	[147]
Poly(L-lactic acid) (PLLA)	L-Arginine (Arg)/hyaluronic acid (HA)	Tendon tissue engineering	[148]

### 3.4. Drug Delivery Systems

Drug delivery systems (DDSs) consist of specific formulations or devices designed to introduce therapeutic agents into the body, regulating their release rate, duration of action, and target sites [149]. These systems enable targeted in situ action, minimizing the drawbacks of systemic exposure associated with free drugs or traditional oral and intravenous administration while also maximizing drug effects through controlled and sustained release at the target site [56].

Electrospun nanofibers, due to their biomimetic properties which closely resemble the extracellular matrix (ECM), promote cell attachment, migration, and proliferation in biological systems, making them ideal candidates for drug delivery and regenerative medicine [150]. By regulating material composition (e.g., hydrophobic and hydrophilic materials), microstructure (e.g., homogeneous, core–shell, and multilayer structures), and macrostructure (e.g., stacked mesh structures), electrospun nanofibers demonstrate significant advantages in precisely controlling drug release rates, allowing for customized drug release profiles [56].

In addition to delivering antibacterial agents, anti-inflammatory drugs, and bioactive molecules, as highlighted in previous sections, electrospun-nanofiber-based drug delivery systems have garnered significant interest in the fields of cancer therapy and gene delivery in recent years [54,151].

Representative applications of electrospun nanofibers in cancer therapy, as illustrated in Table 5, demonstrate their potential. In cancer treatment, electrospun nanofibers can achieve controlled local release of anticancer agents (e.g., chemotherapeutic drugs), significantly improving therapeutic efficacy while reducing systemic toxicity [152]. Beyond chemotherapy [153], electrospun nanofibers have recently been successfully applied in other cancer treatment modalities, including magnetic hyperthermia [154], photothermal therapy [155], and photodynamic therapy [156]. Interestingly, these modalities can also be combined, such as integrating chemotherapeutic drugs with photothermal therapy, to synergistically treat malignant tumors and achieve more pronounced antitumor effects [157,158,159]. For example, in Martorana et al.’s study, redox-responsive glutathione-extended polyurethane urea (PolyCEGS) was used to prepare electrospun membranes loaded with paclitaxel (PTX) and gold nanorods (AuNRs), achieving a combined redox/near-infrared (NIR) light-responsive release of chemotherapy and thermal effects, resulting in significant synergistic cytotoxicity in human colon cancer and breast cancer cell lines [158].

Gene therapy, which delivers genetic materials and gene-editing tools via viral and non-viral vectors to regulate cellular protein expression and function, holds the potential to repair genetic defects, suppress harmful genes, or activate specific genes, offering new strategies for cancer and tissue injury treatment.

By manipulating gene transcription and translation processes, gene therapy can inhibit or restore the function of specific genes, thereby attenuating the malignant characteristics of cancer cells [160]. Zhang et al. [161] developed a novel dual-release system using liposomes to encapsulate minichromosome maintenance protein 4 (MCM4)-siRNA and metal–organic frameworks (MOFs) to deliver the chemotherapeutic drug cisplatin, co-loaded onto electrospun PLA membranes. Both in vitro and in vivo results confirmed that this membrane system effectively delivers therapeutic MCM4-siRNA and releases cisplatin to inhibit melanoma growth.

Additionally, combining electrospinning technology with gene therapy for tissue injury repair has become an innovative and promising research direction. Sun et al. [162] used a microfluidic chip to transform recombinant IL-10 plasmid (pIL-10) into lipid nanoparticles (LNPs) (pIL-10-LNP), which were then uniformly dispersed in an HA solution using microsol electrospinning. The outer layer was encapsulated with PLA to protect the target gene and incorporate it into the nerve growth factor (NGF). By balancing the inflammatory response in spinal cord injury (SCI) and promoting the repair and regeneration of neural tissue, this approach improved motor function post-SCI, as demonstrated in rat experiments. In another study, COX-2 was targeted to inhibit inflammatory responses, and a bilayer-structured positively charged micro-nano electrospun fiber membrane was prepared by using electrospinning. This enabled the unidirectional delivery of COX-2 siRNA-loaded cationic nanocarriers, achieving unidirectional gene therapy at the tendon–paratenon interface. Under the charge repulsion of the positively charged layer, more cationic COX-2 siRNA nanocarriers accumulated in the paratenon tissue, enhancing the bioavailability of the gene drug to prevent paratenon adhesion while avoiding adverse effects on the tendon’s fragile endogenous healing process [163].

**Table 5:** Electrospun nanofibers which are used in drug delivery systems for cancer therapy.

Electrospun Polymers	Functional Additives	Therapy Mode	Cancer Type	Ref.
Poly(glycolide-ε-caprolactone)(PGCL)/poly(lactide-glycolide) (PLGA)	Paclitaxel (PTX)/docetaxel (DTX)	Chemotherapy	Prostate cancer	[164]
Polyvinyl alcohol (PVA)	Paclitaxel (PTX)	Chemotherapy	Cervicovaginal cancer	[153]
Polycaprolactone (PCL)	Irinotecan	Chemotherapy	Pancreatic cancer	[165]
Poly[(d,l)-lactide-co-glycolide] (PLGA)	Imiquimod/metronidazole	Chemotherapy	Cervical cancer	[166]
Cellulose acetate (CA)/poly (ethylene oxide) (PEO)	Disulfiram	Chemotherapy	Breast cancer/colon cancer	[167]
Poly(L-lactic acid) (PLLA)	Bismuth selenide (Bi_2_Se_3_) nanoplates	Photothermal therapy	Skin fibroblast	[168]
Polycaprolactone (PCL)	Graphene oxide (GO)	Photothermal therapy	Breast cancer	[169]
poly (lactic acid) (PLA)/polycaprolactone (PCL)	Copper(I) sulfide (Cu_2_S)	Photothermal therapy	Skin cancer	[170]
Silk fibroin (SF)/poly (lactic-co-glycolic acid) (PLGA)	Black phosphorus (BP)	Photothermal therapy	Liver cancer	[155]
Poly(ε-caprolactone) (PCL)/poly(p-dioxanone)	Albumin–Ce6–MnO2 nanoparticles (ACM NPs)	Photodynamic therapy	Oesophageal cancer	[156]
Chitosan (CS)	SGP Photosens (PS)	Photodynamic therapy	Breast cancer	[171]
Polycaprolactone (PCL)	Doxorubicin (DOX)/curcumin (CUR)/Magnetic nanoparticles (MNPs)	Combination of chemotherapy and magnetic thermal therapy	Breast cancer	[172]
Polycaprolactone (PCL)	Doxorubicin (DOX)/iron oxide nanoparticles (IONPs)	Combination of chemotherapy and magnetic thermal therapy	Cervical cancer	[154]
Gelatin (Gel)	Dihydromyricetin (DMY)/Copper Sulfide (CuS)	Combination of chemotherapy and photothermal therapy	Liver cancer	[157]
Polyurethane urea (PolyCEGS)	paclitaxel (PTX)/gold nanorods (AuNRs)	Combination of chemotherapy and photothermal therapy	Breast cancer/colon cancer	[158]
Poly(ε-caprolactone) (PCL)/poly (D,L-lactic-co-glycolic acid) (PLGA)	Doxorubicin (DOX)/pyrrole	Combination of chemotherapy and photothermal therapy	Breast cancer/colon cancer	[173]
Polycaprolactone (PCL)/polylactic acid(PLA)	Curcumin (CUR)/polydopamine (PDA)	Combination of chemotherapy and photothermal therapy	Oral squamous cell carcinoma	[159]
Hydroxypropyl methylcellulose (HPMC)/Eudragit L100-55	Carmofur (CAR)/rose bengal (RB)	Combination of chemotherapy and photodynamic therapy	Colon cancer	[174]
Polycaprolactone (PCL)	Natural melanin nanoparticles (MNPs)	Combination of chemotherapy and photodynamic therapy	Malignant melanoma	[175]

### 3.5. Sensors

Sensors have been widely applied in fields such as medicine, clinical diagnostics, and environmental monitoring. A sensor consists of a receptor and a transducer. The receptor converts the information to be analyzed into a measurable form of energy, which is then transformed into a measurable signal by the transducer [176]. In recent years, electrospun nanofibers have been proposed as an alternative for sensor fabrication due to their unique characteristics. Sensors made from electrospun nanofibers exhibit higher loading capacity, better sensitivity, and faster response times, effectively enhancing their sensing performance. By incorporating functional components (e.g., metal oxides, carbon nanotubes, conjugated polymers) into electrospun fibers the synergistic effects between different materials can be leveraged to improve the catalytic activity and sensitivity of sensors, expanding the application range of electrospun nanofibers [177,178].

#### 3.5.1. Applications of Sensors in Biomarker Detection

Traditional biomarker detection methods, including enzyme-linked immunosorbent assay (ELISA), mass spectrometry, chromatography, and other technologies, have various limitations, including high equipment requirements, complex operation, and the inability to monitor in real time. In recent years, scientists have been continuously exploring new, more sensitive and efficient biomarker detection methods, and the fabrication of biosensors based on electrospinning technology is considered to be one of the most promising methods [179].

In cancer diagnosis and prognosis evaluation, nanofiber sensors fabricated via electrospinning primarily identify cancer-related tumor markers. Paimard et al. [180] developed an electrochemical immunosensor for CEA biomarker detection, modifying electrospun nanofibers with gold nanoparticles (GNPs) and multi-walled carbon nanotubes (MWCNTs), followed by immobilizing CEA antibodies on the electrode surface. Under optimal conditions, the immunosensor exhibited high sensitivity for CEA biomarkers in the low concentration range of 0.4–125 ng mL^−1^, with a detection limit of 0.09 ng mL^−1^ (S/N = 3).

Cardiac troponin I (cTnI), as a characteristic biomarker, plays a crucial role in the diagnosis of cardiovascular diseases. Gobalu et al. [181] proposed a molybdenum disulfide/cellulose acetate (MoS_2_/CA) nanofiber composite on a screen-printed electrode for the impedance spectroscopic detection of cTnI. This electrochemical nano-biosensor could detect troponin I up to 10 fM, with 90% stability after 6 weeks and approximately 5-times-higher selectivity compared to other proteins. The sensor’s sensitivity was 0.89 μA mM^−1^ cm^−2^, with an RSD value of 3.8%.

For neurological diseases, current electrospun biosensors are advancing toward early diagnosis and monitoring. Liu et al. [182] developed an electrochemical DNA biosensor based on polyamide/polyaniline carbon nanotubes (PA/PANI-CNTs) for the rapid detection of Aβ42 protein in human blood, enabling early diagnosis of Alzheimer’s disease. PANI and CNTs not only exhibit high conductivity but also provide abundant binding sites for Aβ42 aptamers. After immobilization, the aptamer could precisely and specifically detect Aβ42 in human blood within 4 min, with a rapid response, low detection limits, and a wide linear detection range.

Monitoring metabolic diseases can be achieved by sensing key biomarkers of the metabolic system, such as glucose and lactate. Xiao et al. [183] achieved dual sensing of glucose and pH by incorporating metal oxides into electrodeposited gold (Au). By constructing a p-n heterojunction to form a Schottky interface the sensitivity of glucose detection was improved, reaching 3.52 μA mM^−1^ cm^−2^, with a detection limit of 20 μM and a linear range of up to 3 mM. In a study by Yang et al. [184], a TiO_2_@ZnO lactate sensor was prepared using electrospinning and hot-pressing techniques. The bead-like heterojunction exhibited significant charge transfer activity, photocurrent density, and minimal overpotential, demonstrating a low detection limit of 0.031 μM, a high sensitivity of 29.18 μA mM^−1^, a wide linear range between 0.1 and 1.0 mM, and excellent reproducibility.

#### 3.5.2. Applications of Sensors in Wearable Devices

Wearable devices refer to microelectronic devices worn directly on the skin to sense physical and chemical signals or provide convenient human–machine interaction. These devices facilitate continuous health monitoring and can replace bulky, complex traditional equipment [185].

Sharma et al. [186] proposed an omnidirectional strain-sensitive stretchable electronic glove sensor with functionalities for pressure, temperature, humidity, and electrocardiogram sensing, while minimizing crosstalk. This wearable sensor features a wavy sensing region and interconnect design that stretches according to applied deformation without affecting sensor performance, offering full mechanical stretchability and remote data transmission to users. In a study by Choi et al. [187], a novel composite film—the polybutadiene-based polyurethane (PBU)/AgNW/PBU sensor (PAPS)—was fabricated, demonstrating exceptional mechanical stability and accuracy in motion detection. When attached to different parts of the human body, the PAPS generated unique signal curves reflecting specific body parts and motion levels, with signal interpretation achieved through machine learning and deep learning algorithms.

### 3.6. Other Special Functions

Various kinds of special functional nanofibers have been widely used in clothing textiles [188], food packaging [189], pollutant treatment [190], and electronic device production [191]. This section will introduce electrospun nanofibers with special functionalization, including UV protection, antistatic properties, water resistance, electromagnetic interference shielding, and catalytic functionality.

Achieving UV-shielding functionality while maintaining comfort is critical for textiles, from conventional fabrics to electrospun nanofibers, necessitating ongoing material innovations. Liu et al. [192] prepared PVDF/F-TiO_2_/UV9, a waterproof and breathable nanofiber membrane with excellent UV resistance. By adding F-TiO_2_ nanoparticles, the waterproofing performance of the PVDF nanofiber membrane was significantly improved, while achieving durable UV resistance. The UV resistance was further enhanced by using the organic UV absorber UV9. The optimized ratio of PVDF/F-TiO_2_/UV9 gives it moisture permeability (10.6 kg m^−2^ d^−1^), water resistance (60.2 kPa), tensile strength (15.1 MPa), and an excellent UV protection factor (UPF value of 1690.7), which makes it suitable for the design of wearable textile products for outdoor protective clothing, healthcare, and military products.

Antistatic properties are of great importance in electronic device applications. Good antistatic properties will increase the reliability of the equipment and extend its service life. Xing et al. [193] prepared new antistatic composite nanofibers of PVDF/RTIL via electrospinning. The addition of RTIL increased the average fiber diameter and rough fiber surface of the PVDF/RTIL nanofibers and provided excellent ductility and conductivity, which made the composite nanofibers superb antistatic fiber materials. In addition, RTIL significantly enhances the hydrophobicity of PVDF fibers. These excellent properties make PVDF/RTIL a promising candidate for micro- and nano-electronic device applications.

Electrospun nanofibers are characterized by their adjustable pore structure, which allows for the precise control of wettability. These materials exhibit an excellent waterproof performance while facilitating gas permeation, making them highly versatile for a range of applications. Yao et al. [194] used electrospinning to prepare a liquid dehumidifying dual-layer nanofiber membrane (DLNM) with directional vapor-phase transport and water repellency through PVDF. The nanoporous structure and rough surface of the PVDF nanofiber membrane provided water repellency for the DLNM, and the water vapor permeability coefficient of the membrane was as high as 539.67 g·μm^2^·24 h·Pa. This innovative technique offers a novel approach to directional vapor-phase transport and the construction of waterproof membranes.

Some electrospun nanofibers have been found to possess excellent electromagnetic interference (EMI)-shielding properties. These materials prevent external EMI from affecting the normal operation of equipment and reduce the EMI radiation generated by the equipment itself, thereby minimizing pollution of the surrounding environment. A lightweight and flexible thermally stabilized composite was prepared by combining silica nanofibrous membranes (SNMs) with MXene@c-MWCNT hybrid membranes developed by Han et al. [195]. The combination of an SNM and an MXene@c-MWCNT6:4 monolayer (SMC1) with PVA solution resulted in a solution with a low thermal conductivity (0.066 W m^−1^ K^−1^) and good EMI shielding properties (average EMI SET, 37.8 dB). As the number of functional unit layers increases, the overall thermal insulation performance of the entire composite film remains stable and the EMI-shielding performance is greatly improved (with the average EMI SET increasing to 55.4 dB for three-unit layers).

Hierarchically structured nanomaterials play an important role in catalysis. The electrospinning technique is capable of producing nanofibers with different types of complex structures to achieve stable and efficient catalytic performance. Guo et al. [196] designed a one-dimensional confined nanoreactor with a heterogeneous core–shell structure via the customized encapsulation of Z-type heterojunction CuS quantum dots/BiVO_4_ (CuS QDs/BiVO_4_) and Y_2_O_2_S-Er, Yb, realizing a synergistic effect of efficient catalysis and temperature monitoring. The high surface energy of the quantum dots and the efficient electron transport channel at the heterogeneous interface enable the nanoreactor to have both an excellent catalytic performance and monitorable photocatalysis. In addition, the reactor confines the reaction to different enclosed spaces, increasing effective intermolecular collisions, facilitating the catalytic process, and circumventing potential unknown interaction effects.

## 4. Challenges and Perspectives

This review systematically examines functionalized electrospun nanofibers (FENFs), focusing on critical electrospinning parameters, primary functionalization strategies, and their diverse biomedical applications—such as in antibacterial uses, anti-inflammatory therapies, tissue repair and regeneration, drug delivery systems, biosensing, and specialized functionalities.

However, the practical application of FENFs still faces several substantial challenges.

Controllability: Minor fluctuations in the solution, process, and environmental conditions can significantly influence the morphology and diameter of fibers, thereby critically affecting the consistency of material properties. Currently, the optimization of process parameters mainly relies on empirical trial-and-error approaches, lacking systematic theoretical guidance and intelligent control methods [29,197].

Stability: The use of high voltage and organic solvents during electrospinning, as well as long-term storage, may lead to the inactivation of functional materials, such as growth factors, genes, and enzymes [198]. Numerous studies have developed stimuli-responsive electrospun nanofibers by incorporating materials responsive to light, heat, pH, or electricity. However, the complexity of the biological environment often makes it challenging to achieve precise control over their release behavior [199].

Safety: The adverse effects of organic solvents and their residues on biological systems and the environment cannot be overlooked [200,201]. Furthermore, the biocompatibility and potential toxicity of polymers require systematic investigation. For instance, the acidic byproducts of aliphatic polyesters can potentially trigger inflammatory responses and foreign body reactions in biological systems, posing challenges for biomedical applications [202,203].

Clinical Translation and Large-Scale Production: Traditional sterilization methods, such as high-temperature and high-pressure treatments, may compromise the structural integrity of nanofibers and the functionality of bioactive materials [204,205]. Conventional electrospinning technology typically achieves a production rate of 0.01^−1^ g/h by fiber weight or 1.0–5.0 mL/h by flow rate, which falls significantly short of industrial-scale production requirements. Moreover, the industrial production cost is a key factor restricting its application. For example, the maintenance cost of multi-needle electrospinning equipment is relatively high, and the introduction of green solvents may increase raw material expenses [206,207].

Based on current research progress and existing challenges, the future development of FENFs can be summarized in the following directions:

Intelligent Manufacturing: Using advanced technologies, such as artificial intelligence (AI), when fabricating FENFs may be the solution to improving controllability and reproductivity. For instance, given that morphology can affect drug solubility, an AI model based on convolutional neural networks (CNNs) was developed to examine this characteristic and detect manufacturing defects. Camera images and trained AI models were used to measure the diameter of electrospun fiber samples, enabling the rapid analysis of fiber diameter with results comparable to those obtained from scanning electron microscopy (SEM) [208].

Fabrication Process Optimization: Electrospinning process optimization should ensure a balance between eco-friendliness and industrial applicability. To address environmental concerns, it is crucial to use biodegradable polymers, green solvents, and high-efficiency equipment, despite the increased costs. Suspension electrospinning, also called green electrospinning, is a novel alternative for overcoming these issues. Using this technology, hydrophobic polymers can be spun at higher concentrations than the ones used in solution electrospinning, using water as an electrospinning medium [209].

Multifunctional Integration: Co-loading multiple functional materials into electrospun nanofibers can achieve synergistic effects. Personalized design and precise construction can also be realized through its combination with other technologies, such as 3D printing, electrospraying, self-assembly, plasma treatment, chemical crosslinking, and surface patterning. Additionally, next-generation smart nanofibers may integrate multi-responsive systems, utilizing biosensors and feedback control mechanisms to precisely modulate release behaviors in complex biological environments [210].

## Figures and Tables

**Table 1:** Electrospinning parameters and their effects on other parameters and nanofiber morphology (considering increases within optimal ranges). ↑: increase, ↓: decrease.

Parameters	Effects on Other Parameters	Effects on Nanofiber Morphology
Polymer concentration	Viscosity ↑ Surface tension ↑	Diameter ↑ Mechanical strengh ↑
Molecular weight of polymer	Viscosity ↑ Surface tension ↓ Jet stability ↑	Bead formation ↓
Conductivity	Dielectric constant ↑ Charge density ↑	Diameter ↓
Viscosity	G’↑ G” ↑ G’/G” ↓	Diameter ↑ Bead formation ↓
Surface tension	Jet stability ↑	Bead formation ↑
Applied voltage	Size of Taylor Cone ↑ Charge density ↑	The effect on diameter is under debate.
Flow rate	-	Diameter ↓
Collecting distance	Evaporation rate ↓ Electric field intensity ↓	Diameter ↑ Fiber uniformity ↓
Temperature	Viscosity ↓	Diameter ↓
Relative humidity	Evaporation rate ↓	The effect on diameter relies on polymer/solvent system. Porosity ↑ Bead formation ↑

**Table 2:** Electrospun nanofibers which are used in wound dressings and food packaging.

Electrospun Polymers	Antibacterial Additives	Types of Additives	Functionalization Methods	Application Area	Ref.
Poly(lactic acid) (PLA)/poly(ethyleneoxide) (PEO)	Silver nanoparticles (Ag NPs)	Inorganic namomaterial	Blend electrospinning	Wound dressing	[83]
Chitosan (CS)/polycaprolactone (PCL)	Zinc oxide nanoparticles (ZnO NPs)	Inorganic namomaterial	Co-axial electrospinning	Wound dressing	[84]
Polycaprolactone (PCL)/poly(acrylic acid) (PAA)	Graphene oxides (GOs)	Inorganic namomaterial	Blend electrospinning/in situ polymerization	Wound dressing	[85]
Gelatin(Gel)/chitosan (CS)	Mxenes	Inorganic namomaterial	Blend electrospinning	Wound dressing	[86]
Polycaprolactone (PCL)	Covalent organic frameworks (COFs) Curcumin (CUR)	Organic nanomaterial/natural-derived compounds	Blend electrospinning	Wound dressing	[87]
Cellulose Acetate (CA)	Chitosan nanoparticles (CS NPs)/Eucalyptus Oil	Organic nanomaterial/natural-derived compounds	Blend electrospinning	Wound dressing	[88]
Chitosan (CS)	Zeolitic Imidazolate Framework-8 (ZIF-8)	Inorganic-organic hybrid nanomaterial	In situ growth	Wound dressing	[89]
Polycaprolactone (PCL)	Porous coordination network−224 (PCN−224)	Inorganic-organic hybrid nanomaterial	Blend electrospinning	Wound dressing	[90]
Gelatin (Gel)	MIL-100(Fe)@IR775	Inorganic-organic hybrid nanomaterial	Blend electrospinning	Wound dressing	[91]
Gelatin (Gel)	Curcumin (CUR)/borneol	Natural-derived compounds	Blend electrospinning	Wound dressing	[92]
Polycaprolactone (PCL)	Manuka honey/essential oils	Natural-derived compounds	Layer-by-layer assembly	Wound dressing	[93]
Poly(lactic acid) (PLA)	AMP	Biofunctional agents	Blend electrospinning	Wound dressing	[94]
Zein/polyethylene oxide (PEO)	Resveratrol (RE)/Ag NPs	Inorganic namomaterial/natural-derived compounds	Co-axial electrospinning	Food package	[95]
Starch/(PVA)	Ag-ZrP	Inorganic namomaterial	Blend electrospinning/crosslinking	Food package	[96]
Chitosan (CS)/polyethylene oxide (PEO)	Thymol (Thy)	Natural-derived compounds	Co-axial electrospinning/in situ crosslinking	Food package	[97]
Polyvinyl Butyral (PVB)	Camellia oil (CO)/ZnO-TiO_2_ composite nanoparticles (ZT)	Inorganic namomaterial/natural-derived compounds	Blend electrospinning	Food package	[98]
Poly(lactic acid) (PLA)	Cinnamaldehyde (CMA)/tea polyphenol (TP)	Natural-derived compounds	Co-axial electrospinning	Food package	[99]
Poly(lactic acid) (PLA)	Octyl gallate (OG)	Natural-derived compounds	Blend electrospinning	Food package	[100]
Poly(lactic acid) (PLA)	Wormwood Oil	Natural-derived compounds	Blend electrospinning	Food package	[101]
Chitosan(CS)/polyvinyl alcohol (PVA)	Fe_3_O_4_	Inorganic namomaterial	Blend electrospinning	Food package	[102]
Cellulose Acetate (CA)	NiO	Inorganic namomaterial	Blend electrospinning	Food package	[103]

**Table 3:** Electrospun nanofibers which are used for anti-inflammation.

Electrospun Polymers	Anti-Inflammatory Materials	Anti-Inflammatory Strategy	Functionalization Methods	Application Area	Ref.
Polycaprolactone (PCL)/gelatin (Gel)	4-Octyl itaconate (OI)/chitosan (CS)	Direct strategy	Blend electrospinning/covalent grafting	Wound healing	[111]
Poly (lactic acid) (PLA)	Puerarin (Pue)	Direct strategy	Blend electrospinning	Pelvic floor reconstruction	[112]
Gelatin methacryloyl (GelMA)	Naringenin (NA)	Direct strategy	Blend electrospinning	Bone tissue engineering	[113]
ZnO-SiO_2_/chitosan (CS)	Aspirin (ASA)	Direct strategy	Blend electrospinning/crosslinking	Bone tissue engineering	[114]
Gelatin methacryloyl (GelMA)	Casein enzymatic hydrolysate (CEH)	Direct strategy	Blend electrospinning/crosslinking	Vital pulp therapy	[115]
Polycaprolactone (PCL)	Prussian blue nanocrystals (PBNCs)/heparin sodium (Hep)	Direct strategy	Electrospraying/chemical deposition	Wound healing	[116]
Polyvinyl alcohol (PVA)/sodium alginate (SA)	Ag-Hes NPs (using hesperidin as reducing and capping agen)	Indirect strategy	Blend electrospinning	Wound healing	[117]
Polyethylene oxide (PEO)/hydroxypropylmethylcellulose (HPMC)	Chloramphenicol (CAM)/beta-glucan (βG)/chitosan (CHI)	Indirect strategy	Blend electrospinning	Wound healing	[118]
Poly(ε-caprolactone) (PCL)	Thymol (THY)/tyrosol (TYR)	Indirect strategy	Blend electrospinning	Wound healing	[119]
Poly(3-hydroxybutyrate-co-4-hydroxybutyrate) (P34HB)	Ciprofloxacin (CIP)/dimethyloxalylglycine (DMOG)	Indirect strategy	Blend electrospinning	Wound healing	[120]
Polyvinylpyrrolidone (PVP)/chitosan (CS)	Dihydromyricetin (DHM)	Indirect strategy	Blend electrospinning	Wound healing	[121]
Polyvinyl alcohol (PVA)/poly(ε-caprolactone) (PCL)	polyhexamethylene guanidine hydrochloride (PHGC)/hydrophobic indomethacin (Indo)	Synergetic strategy	Blend electrospinning/bidirectional electrospinning	Wound healing	[122]
Poly(esterurethane)urea (PEUU)/silk fibroin (SF)	Magnolol (Mag)	Synergetic strategy	Blend electrospinning/post-hydrogen-bond crosslinking	Wound healing	[123]
Poly(ε-caprolactone) (PCL)/polydopamine (PDA)	ε-polyL-lysine (ε-PL)/ibuprofen (IBU)	Synergetic strategy	Co-axial electrospinning/PDA-assisted assembly	Wound healing	[124]
Poly (lactic acid) (PLA)	Sulfated chitosan (SCS)/polydopamine (PDA)/gentamicin (GS)	Synergetic strategy	Blend electrospinning/PDA-assisted assembly	Wound healing	[125]

## Data Availability

Not applicable.
